# Advancements in diagnosing oral potentially malignant disorders: leveraging Vision transformers for multi-class detection

**DOI:** 10.1007/s00784-024-05762-8

**Published:** 2024-06-08

**Authors:** Shankeeth Vinayahalingam, Niels van Nistelrooij, René Rothweiler, Alessandro Tel, Tim Verhoeven, Daniel Tröltzsch, Marco Kesting, Stefaan Bergé, Tong Xi, Max Heiland, Tabea Flügge

**Affiliations:** 1 Department of Oral and Maxillofacial Surgery, Radboud University Medical Centre, Nijmegen, the Netherlands; 2 Department of Artificial Intelligence, Radboud University, Nijmegen, the Netherlands; 3 Department of Oral and Maxillofacial Surgery, Universitätsklinikum Münster, Münster, Germany; 4 Department of Oral and Maxillofacial Surgery, Charité – Universitätsmedizin Berlin, corporate member of Freie Universität Berlin and Humboldt- Universität zu Berlin, Hindenburgdamm 30, 12203 Berlin, Germany; 5 Department of Oral and Maxillofacial Surgery, Translational Implantology, Medical Center, Faculty of Medicine, University of Freiburg, University of Freiburg, Freiburg, Germany; 6 Clinic of Maxillofacial Surgery, Head&Neck and Neuroscience Department, University Hospital of Udine, Udine, Italy; 7 Department of Oral and Cranio-Maxillofacial Surgery, Friedrich-Alexander-University Erlangen- Nuremberg (FAU), Erlangen, Germany

**Keywords:** Artificial Intelligence, Deep learning, Oral squamous cell carcinoma, Leukoplakia, Oral lichen planus, Malignant transformation

## Abstract

**Objectives:**

Diagnosing oral potentially malignant disorders (OPMD) is critical to prevent oral cancer. This study aims to automatically detect and classify the most common pre-malignant oral lesions, such as leukoplakia and oral lichen planus (OLP), and distinguish them from oral squamous cell carcinomas (OSCC) and healthy oral mucosa on clinical photographs using vision transformers.

**Methods:**

4,161 photographs of healthy mucosa, leukoplakia, OLP, and OSCC were included. Findings were annotated pixel-wise and reviewed by three clinicians. The photographs were divided into 3,337 for training and validation and 824 for testing. The training and validation images were further divided into five folds with stratification. A Mask R-CNN with a Swin Transformer was trained five times with cross-validation, and the held-out test split was used to evaluate the model performance. The precision, F1-score, sensitivity, specificity, and accuracy were calculated. The area under the receiver operating characteristics curve (AUC) and the confusion matrix of the most effective model were presented.

**Results:**

The detection of OSCC with the employed model yielded an F1 of 0.852 and AUC of 0.974. The detection of OLP had an F1 of 0.825 and AUC of 0.948. For leukoplakia the F1 was 0.796 and the AUC was 0.938.

**Conclusions:**

OSCC were effectively detected with the employed model, whereas the detection of OLP and leukoplakia was moderately effective.

**Clinical relevance:**

Oral cancer is often detected in advanced stages. The demonstrated technology may support the detection and observation of OPMD to lower the disease burden and identify malignant oral cavity lesions earlier.

## Introduction

Diagnosing oral potentially malignant disorders (OPMD) is crucial in dental examinations. It plays a significant role in providing adequate treatment, educating patients about associated risks, and preventing oral cancer. OPMD refers to a group of mucosal lesions with an increased risk of developing into oral squamous cell carcinoma (OSCC). Therefore, accurate diagnosis, careful observation, or timely resection of OPMD are essential to prevent the transformation into oral cancer [1].

Common OPMD conditions include leukoplakia, proliferative verrucous leukoplakia, erythroplakia, oral submucous fibrosis, and oral lichen planus [1, 2] Leukoplakia is among the most frequently encountered OPMD, with a global prevalence of 4,11% [3]. It is diagnosed based on clinical presentation and can be defined as a not wipeable “predominantly white plaque of questionable risk, having excluded other known diseases or disorders that do not carry an increased risk for cancer.” [2].

Oral lichen planus (OLP) is an autoimmune chronic inflammatory disease affecting the skin, oral, esophageal, and vaginal mucosa. In its oral form, OLP typically manifests with a distinctive white reticular pattern and can present in erosive, atrophic, bullous, or plaque-like forms [4]. Differential diagnoses for OLP include frictional keratosis, candidiasis, leukoplakia, lupus erythematosus, pemphigus vulgaris, mucous membrane pemphigoid, and chronic ulcerative stomatitis [5]. The prevalence of OLP is approximately 1.27%, with slightly higher rates in women than men [6, 7].

The transformation rate of OPMD varies significantly, ranging from 1.4 to 49.5%. Predicting the risk of malignant transformation remains challenging due to the lack of accurate diagnostic methods [8]. On average, the transformation rate for OLP is 1.4%, while erythroplakia has an average transformation rate of 21% [9–11]. Notably, patients with proliferative verrucous leukoplakia have a transformation rate of up to 74% [12, 13].

Various methods are employed to diagnose OPMD, including visual inspection supported by vital staining [14], autofluorescence, and reflectance spectroscopy [15]. Invasive approaches such as brush biopsy with cytologic testing and incision biopsy are also utilized [16, 17]. Cytologic testing of suspicious lesions has demonstrated a sensitivity of 0.92 and a specificity of 0.94 for OPMD and OSCC [1]. However, the diagnostic accuracy of autofluorescence, vital staining, and reflectance spectroscopy in identifying OPMD and OSCC is limited [1]. Autofluorescence shows higher sensitivity (0.90) and specificity (0.72) for suspicious lesions compared to innocuous lesions (sensitivity 0.50, specificity 0.39) [1, 18]. Similarly, vital staining and reflectance spectroscopy exhibit relatively low diagnostic accuracies for suspicious lesions [1].

Deep learning models have gained significant traction in medical image analysis. Specifically, convolutional neural networks (CNN) and vision transformer (ViT) models have emerged as powerful tools for detecting pathologies in photographic images. Previous studies assessed the accuracy of CNN models based on images of OSCC and other photos of oral mucosal lesions, including OPMD [5, 19–27] [5, 19]. 

Recognizing the need for alternatives to biopsies performed on subjectively selected areas, the present study investigates the performance of deep learning models in detecting OPMD, specifically leukoplakia and OLP, and OSCC using photographic images of the oral cavity. The study hypothesized that a Mask R-CNN architecture with a Swin Transformer backbone would obtain high accuracy in the multi-class detection of OLP, leukoplakia, and OSCC. By leveraging the capabilities of machine learning models and harnessing the potential of vision transformers, this research aims to advance non-invasive diagnostic methods for OPMD and oral cancer.

## Materials and methods

### Data description

A total of 4,161 clinical photographs were retrospectively collected from the Department of Oral and Maxillofacial Surgery at Charité – Universitätsmedizin Berlin, Germany. These images comprehensively covered various regions of the oral cavity. The dataset included images featuring OPMD, specifically OLP, and leukoplakia and pictures of OSCC covering TNM stages I to IV. The image resolution was consistently maintained at a minimum of 74 pixels per inch (ppi). All image data were anonymized before analysis. This study was conducted according to the Declaration of Helsinki. The Institutional Review Board (EA2/089/22) approved this study on the 19th of May 2022.

### Data annotation

Disorders in the photographs were pixel-wise segmented and labeled as OLP, leukoplakia, or OSCC. Pictures with a different disorder were excluded from this study. Each photo was annotated by different clinicians independently using the DARWIN Version 2.0 software (V7, London, UK). Three clinicians subsequently reviewed and revised all segmented and labeled photographs (TF, DT, TX). The three reviewers have at least ten years of clinical experience and have completed their specialty training. Each clinician was instructed and calibrated in the annotation task using a standardized protocol before the annotation and reviewing process.

### Data pre-processing

**Fig. 1:**
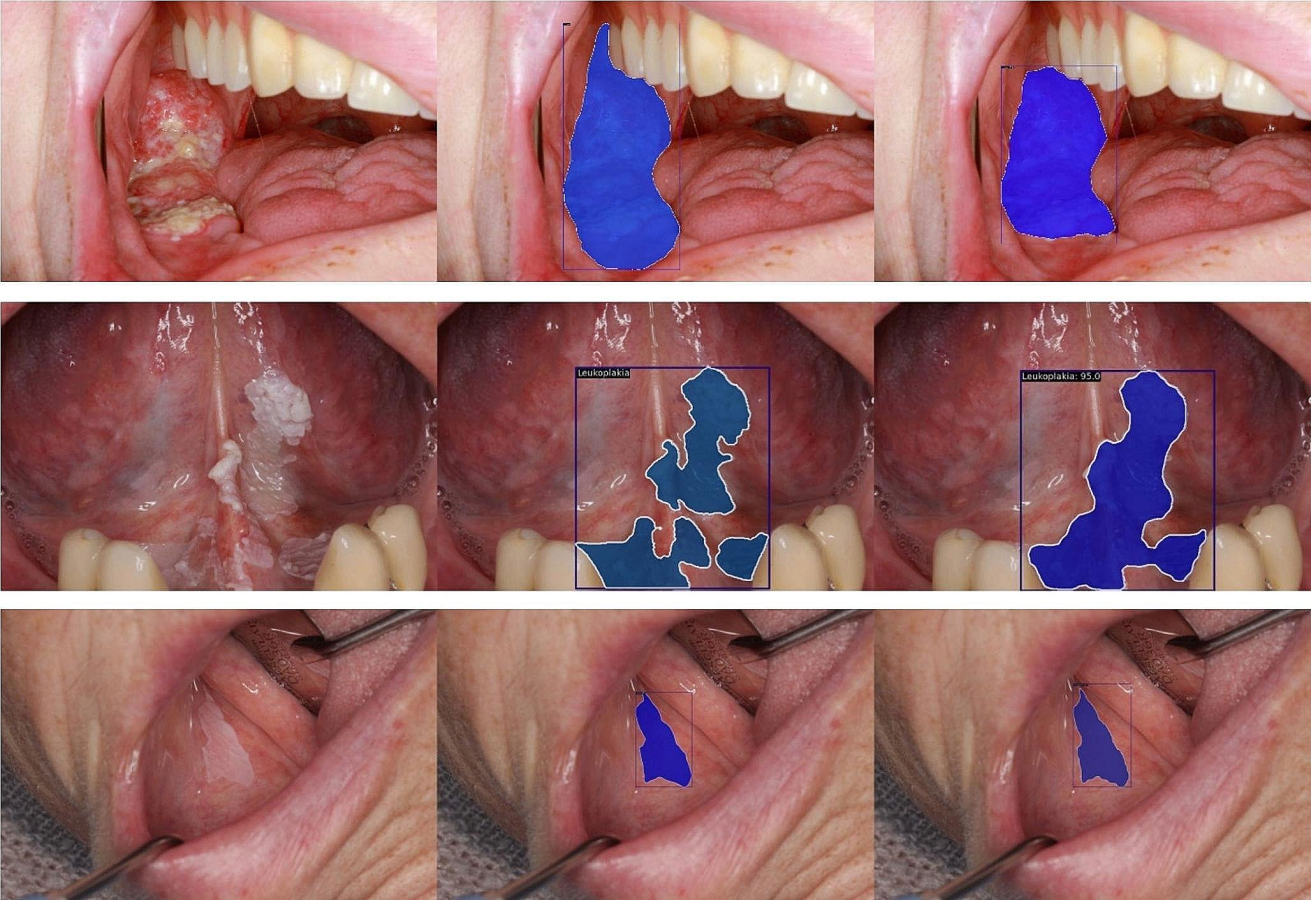
Examples of correct OSCC, leukoplakia, and OLP predictions. The left column is the input image, the middle column is the reference annotation, and the right column is the prediction

The annotated pixels were clustered into objects as connected pixels of the same label. Objects close together were aggregated by merging their annotated pixels (Fig. 1, middle row). More specifically, given a label, all objects of that label were morphologically dilated, and groups of overlapping dilated objects were determined. The original pixels from all objects in a group were merged to form a new object, and the original smaller objects were removed. This was repeated for the three labels (Lichen, Leukoplakia, and OSCC).

### Model architecture

The study employed a Mask R-CNN with a Swin Transformer. Mask R-CNN enhances object detection and instance segmentation, augmenting Faster R-CNN with a mask prediction branch. Swin Transformer’s key attribute is its distinctive window shifting among self-attention layers. This novel technique, connecting windows across layers, efficiently enhances modeling of long-range dependencies. The approach significantly boosts the architecture’s ability to capture intricate patterns and improves the integration of information from various locations within an image.

### Model training

The annotated photographs were divided into 3,337 images for training and validation and 412 images for testing with stratification on the type of disorder, supplemented by a random selection of 412 pictures without an oral disorder. The training and validation images were further divided into five folds with stratification. All images of a patient were strictly grouped either within the test split or within one fold. The Mask R-CNN model was trained five times with cross-validation. The held-out test split was used to evaluate the model performance after training.

The Mask R-CNN model was pre-trained on the COCO dataset and used the small variant of the Swin Transformer as backbone. The model optimization used the AdamW optimizer with a weight decay set 0.05. The training was performed for a maximum of 24 epochs with an initial learning rate of 5e-5 divided by ten after epochs 16 and 22. The Mask R-CNN architecture used the categorical cross-entropy loss function to optimize the classification branch and the L1 loss function to optimize the bounding box regression branch. Images with a rare combination of disorders were oversampled to address class imbalance, and a mini-batch size of 4 was used. The model was implemented in MMDetection based on PyTorch and trained on a single NVIDIA® RTX A6000 48G.

### Model inference

The disorder within a photograph was predicted twice by providing the model with a normal and flipped picture version (test-time augmentation). The image was finally labelled by the disorder segmentation with the highest confidence. If this confidence was below 0.75, the image was labelled as having no disorder.

### Statistical analysis

All models’ predictions on the test photographs were aggregated and compared to the reference annotations using scikit-learn (version 1.3.0). Image-level classification scores were determined for each label by taking the maximum confidence of the model for any predicted object with that label. Classification metrics were reported as follows: $\text{p}\text{r}\text{e}\text{c}\text{i}\text{s}\text{i}\text{o}\text{n} =\frac{TP}{TP+FP}$, $\text{F}1 \text{s}\text{c}\text{o}\text{r}\text{e} =\frac{2TP}{2TP+FP+FN}$(also known as the Dice-coefficient), $\text{r}\text{e}\text{c}\text{a}\text{l}\text{l} =\frac{TP}{TP+FN}$(also known as sensitivity), $\text{s}\text{p}\text{e}\text{c}\text{i}\text{f}\text{i}\text{c}\text{i}\text{t}\text{y} = \frac{TN}{TN+FP}$, and $\text{a}\text{c}\text{c}\text{u}\text{r}\text{a}\text{c}\text{y} =\frac{TP+TN}{TP+TN+FP+FN}$, where TP, TN, FP, and FN denote true positives, true negatives, false positives, and false negatives, respectively. Furthermore, the area under the receiver operating characteristics curve (AUC) of all models and the confusion matrix of the most effective model were presented.

## Results

**Fig. 2:**
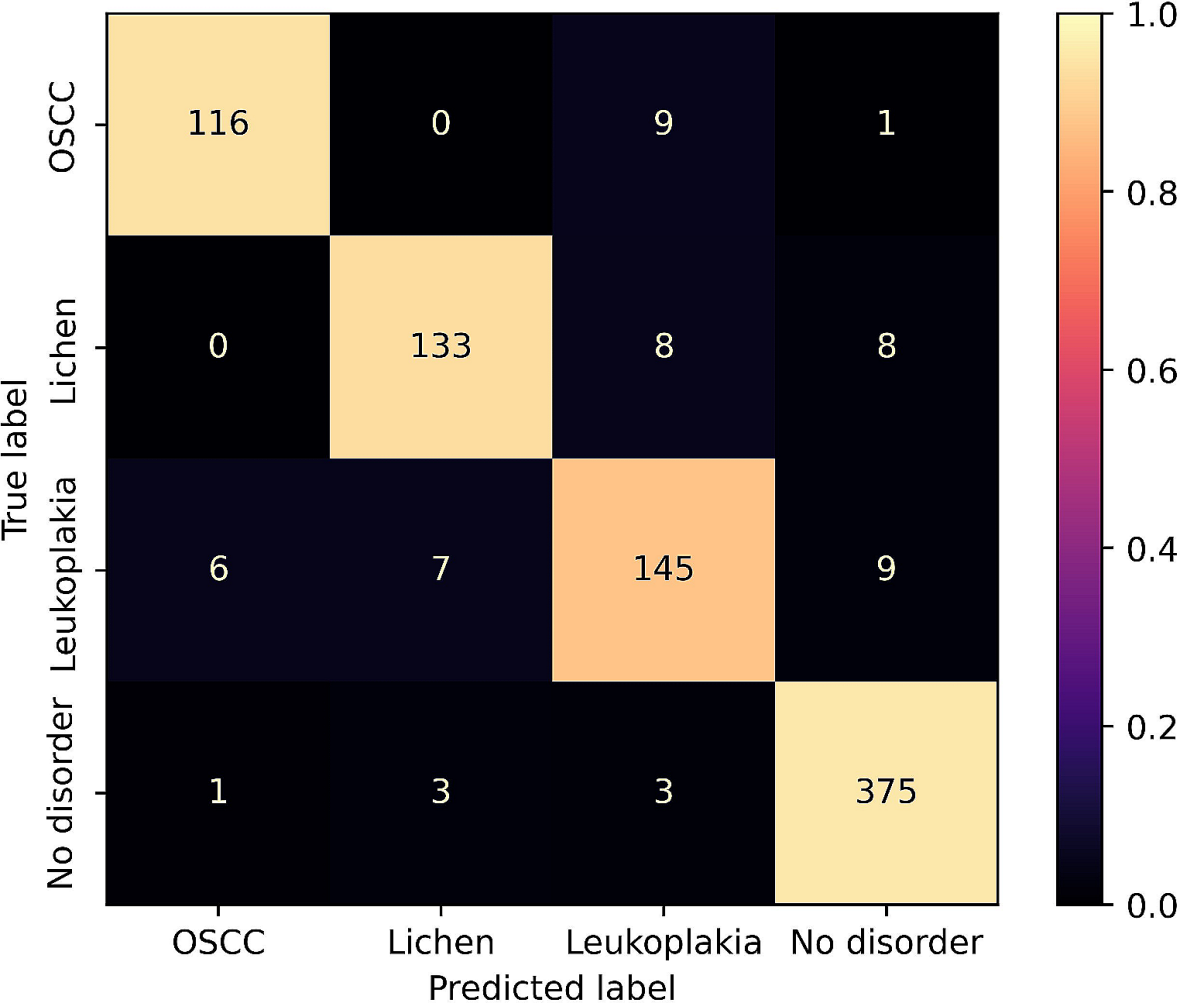
Confusion matrix of the results of multi-class image-level disorder detection. The results of the most effective cross-validation model on the test images are shown. Predicted disorders with a maximum score ≥ 0.75 are matched to reference disorder. The colors are normalized by the number of predicted labels

**Fig. 3:**
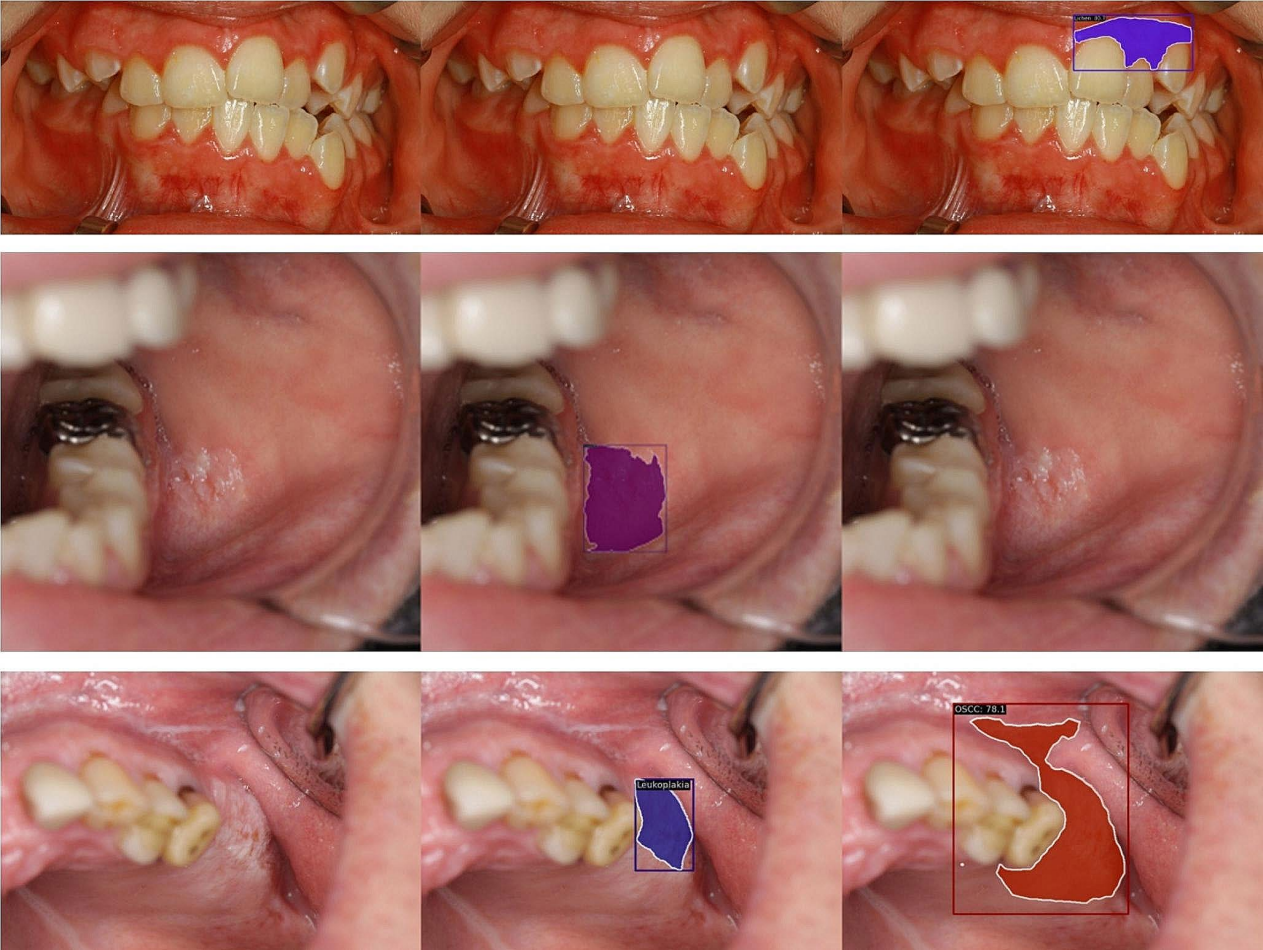
Examples of incorrect predictions. The left column is the input image, the middle is the reference annotation, and the right is the prediction. The scores in the right column are the confidences of the model. The first row illustrates the false-positive prediction. The second row shows the false-negative prediction. The last row represents model’s misclassification

**Fig. 4:**
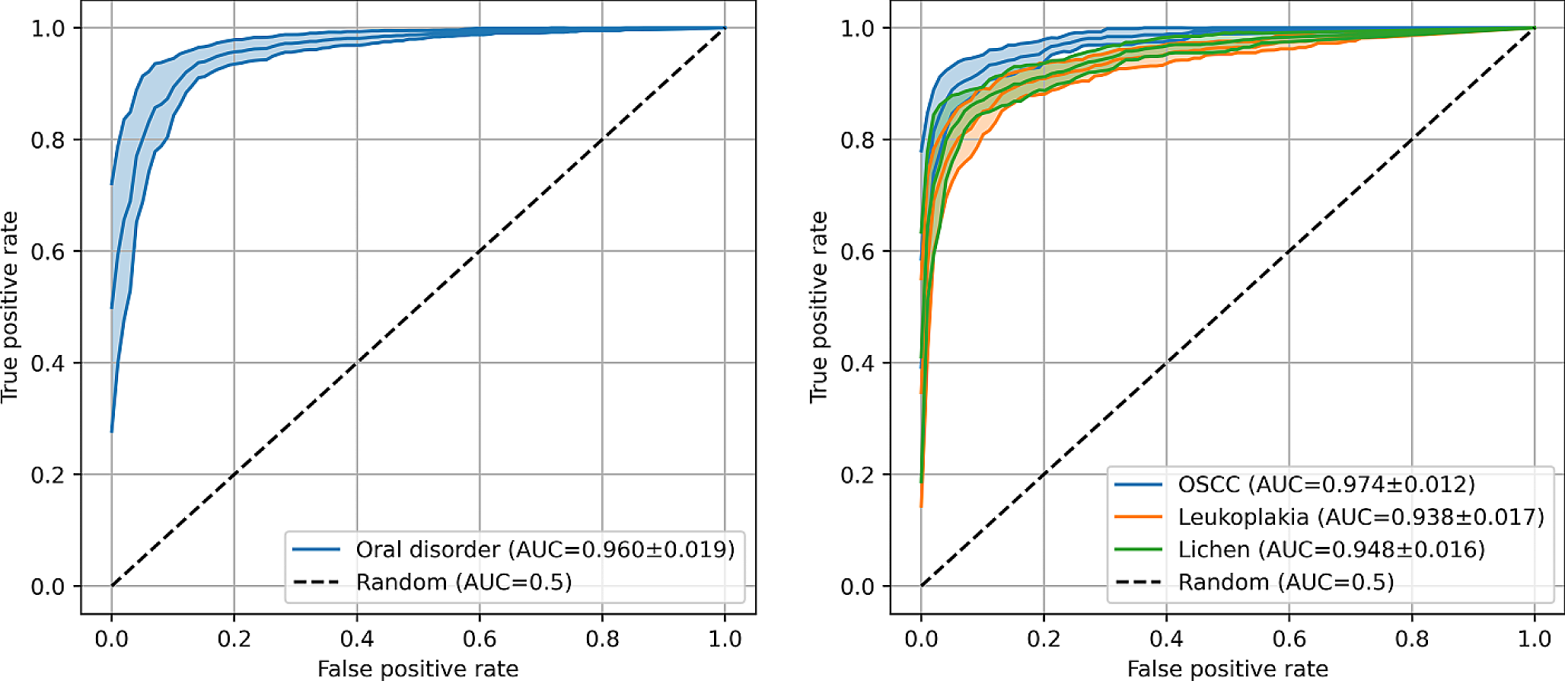
Receiver operating characteristic (ROC) curves of image-level classification. On the left side, the ROC curve illustrates the binary classification (pathology versus no disorder). On the right side, the ROC curve shows the binary classifications for the respective pathologies (OSCC versus no disorder; leukoplakia versus no disorder; OLP versus no disorder). Each center line and peripheral line represent the mean and the mean plus and minus the standard deviation across cross-validation models

The Mask R-CNN model with Swin Transformer backbone effectively detected OSCC (F1 = 0.852, AUC = 0.974). Detection of OLP and leukoplakia disorders was moderately effective (OLP: F1 = 0.825, AUC = 0.948; leukoplakia: F1 = 0.796, AUC = 0.938) (Table 1; Fig. 2). Figures 1 and 3 show that the model could not consistently reproduce the reference segmentations. The image-level predictions often agreed with the reference, as the ROC curves in Fig. 4 depict.

**Table 1:** Classification metrics of multi-class image-level disorder detection on hold-out test set. Mean ± standard deviation is calculated over the five cross-validation models. Predicted disorder with maximum score ≥ 0.75 is matched to reference disorder

	Specificity	Sensitivity	Precision	Accuracy	F1
Lichen	0.976 ± 0.017	0.776 ± 0.053	0.882 ± 0.083	0.940 ± 0.023	0.825 ± 0.064
Leukoplakia	0.954 ± 0.015	0.780 ± 0.048	0.813 ± 0.060	0.919 ± 0.022	0.796 ± 0.053
OSCC	0.975 ± 0.016	0.843 ± 0.039	0.863 ± 0.080	0.955 ± 0.017	0.852 ± 0.053
No disorder	0.877 ± 0.019	00.926 ± 0.041	0.866 ± 0.021	0.900 ± 0.027	0.895 ± 0.029
Average	0.946 ± 0.014	0.831 ± 0.042	0.856 ± 0.054	0.928 ± 0.021	0.842 ± 0.047

## Discussion

OPMD should be accurately diagnosed and treated to minimize patient morbidity and prevent oral cancer. The early detection and treatment of oral cancer are associated with a good prognosis [28]. Nevertheless, the majority of oral cancer cases are detected in an advanced stage, where patients experience symptoms and mucosal changes for several months [29].

The diagnostic accuracy of various non-invasive methods, such as chemiluminescence, autofluorescence imaging, toluidine blue staining, as well as narrow band imaging, ranges from 50.0 to 93.9% for sensitivity and from 12.5 to 94.6% for specificity. However, these examinations are often affected by significant operator-related variability [30]. Consequently, it is concluded that autofluorescence detection and toluidine blue staining are sensitive for detecting oral cancer and malignant lesions but lack specificity [31]. Therefore, employing autofluorescence, tissue reflectance, or vital staining is not recommended to evaluate clinically evident, innocuous, or suspicious oral mucosa lesions. Cytologic adjuncts exhibit slightly higher diagnostic accuracy compared to autofluorescence, tissue reflectance, and vital staining, making them more suitable as screening tools for patients who initially have declined biopsy.

Automatic image analysis has the potential to replace the previous diagnostic aids for OPMD. In recent years, different studies have assessed the potential of AI-based solutions for the early detection of oral cancer and OPMDs [19, 21–27, 32]. In these studies, the datasets were either curated before training to include a specific pathology or healthy mucosa [5, 19], or the multi-class detection did not differentiate between the different OPMDs [20].

The predominant approach in these studies involves using a binary classification model to distinguish between normal mucosa and pathological mucosa. Notably, these studies consistently reported high accuracies with F1-scores above 95% [19, 21–27, 32]. Despite these high metrics, binary classification models should be considered cautiously, as they have potential limitations. Firstly, confronted with a limited dataset for training, these models exhibit a disparity between training and testing accuracy, leading to overfitting issues and a lack of generalizability. Furthermore, the accuracy of binary classifiers is compromised when images are captured from varying angles and backgrounds. Not does the orientation of images influences the diagnostic accuracy, but also the changes in lighting conditions. Binary classification models tend to perform suboptimal when images are taken in more diverse lighting scenarios than the training dataset [33, 34].

To address these limitations, we employed an instance segmentation model. Instance segmentation provides detailed information about object boundaries and localization of individual lesions within an image. Therefore, it allows for counting and differentiation of overlapping lesions. Additionally, instance segmentation models augment the interpretability of the decision-making processes through the use of segmentation masks. In the medical field, generating clear and understandable explanatory structures is crucial to offer clinicians a transparent and explainable system.

The current study utilized a Mask R-CNN model with a Swin Transformer backbone for oral disorder detection. The model demonstrated high accuracies in detecting OSCC with an F1 score of 0.852 and AUC of 0.974, whereas the detection of OLP and leukoplakia disorders was moderately effective, with F1 scores of 0.825 and 0.796, respectively. Despite challenges in reproducing reference segmentations, image-level predictions were often aligned with references, as indicated by ROC curves (Fig. 4). Interestingly, our model encounters challenges similar to those faced by clinicians. The most frequent misclassification for OSCC was Leukoplakia (*n* = 9, Fig. 2), potentially attributed to including Leukoplakia with dysplasia and early-stage OSCC. Clinicians may find it challenging to differentiate these findings, a difficulty also exhibited by the model [22]. Additionally, the misclassification of Leukoplakia as OLP (*n* = 7), previously noted by McParland and Warnakulasuriya, underscores the complexity of differentiation. In a cohort of 51 patients later diagnosed with proliferative verrucous leukoplakia, the initial clinical examination diagnosed OLP in 30 patients [35].

Future research should include multi-center image data representing diverse populations and clinical representations of OPMD. Furthermore, annotations from diverse clinicians will help training more robust algorithms. The application of the models in clinical settings is the next step to research the impact of early detection of OPMD and oral cancer.

## Conclusions

OSCC were effectively detected with the employed deep learning model whereas the detection of OLP and leukoplakia was moderately effective. The automatic detection of OPMD and OSCC through clinical photographs may facilitate precise diagnosis to initiate appropriate treatment.

## Data Availability

No datasets were generated or analysed during the current study.
